# Transcriptome profiling reveals key regulatory factors and metabolic pathways associated with curd formation and development in broccoli

**DOI:** 10.3389/fpls.2024.1418319

**Published:** 2024-07-12

**Authors:** Yinxia Zhu, Ce Liu, Mengyao Zhao, Yuxuan Duan, Jingjing Xie, Chunguo Wang

**Affiliations:** ^1^ College of Life Sciences, Nankai University, Tianjin, China; ^2^ Cucumber Research Institute, Tianjin Academy of Agricultural Sciences, Tianjin, China; ^3^ State Key Laboratory of Vegetable Biobreeding, Tianjin, China

**Keywords:** broccoli, curd, phytohormones, photosynthesis, starch metabolism

## Abstract

Broccoli, a cruciferous vegetable, has a unique indeterminate inflorescence structure known as curds. It is the main edible organ of broccoli and has a rich nutritional value and health benefits. However, the formation and development mechanism of the curd is still not well understood. In the present study, the shoot apical meristem (SAM) stage and three different development stages of curd (formation stage (FS), expansion stage (ES), and maturation stage (MS)) were identified and subjected to transcriptome sequencing to uncover the potential genes and regulatory networks involved in curd formation and development. The results indicated that the genes associated with the development of SAM such as *BolAP1A*, *BolAP1C*, *BolCAL*, and *BolAGL6* play an important role in the abnormal differentiation of the curd apical buds. The genes, *BolFRI*, *BolbHLH89*, *BolKAN4*, *BolAGL12*, and *BolAGL24*, displayed significantly differential expression patterns in curd development may function in the regulation of the transition from inflorescence meristem (IM) to floral meristem (FM). Moreover, gene ontology (GO) and Kyoto Encyclopedia of Genes and Genomes (KEGG) enrichment analysis of the differentially expressed genes (DEGs) indicate that phytohormones, such as auxin (AUX), gibberellins (GA), and abscisic acid (ABA) also play an important role in SAM proliferation and the transition from SAM to IM. In addition, the genes regulating photosynthetic reaction (*BolLHCA1*, *BolLHCB1*, *BolPsbO*, etc.) have a key involvement in the differentiation of secondary IMs during curd expansion. The genes associated with the metabolism of starch and sucrose (e.g., *BolSPS4*, *BolBAM4*) were significantly upregulated at the MS should contribute to the maturation of the curd. These findings provide new insights into the potential key regulatory factors and metabolic pathways involved in the formation and development of broccoli curds.

## 1 Introduction

Broccoli (*Brassica oleracea* var. *Italica*) belongs to the cruciferous family, is characterized by its unique indeterminate inflorescence structure, composing numerous short branches known as curds (Kieffer et al., 1998; Cao et al., 2020). Curd is the main edible organ of broccoli and is rich in various active ingredients and nutrients like phenolic compounds, glucosinolates, vitamins and minerals (Li et al., 2022). Several researches have explored the formation and development mechanism of the curd (Smyth, 1995; Kieffer et al., 1996). However, due to its complex regulatory relationships and the influence of environmental factors, understanding in curd formation and development is still relatively limited (Rosen et al., 2018). As the reproductive developmental organs of broccoli, the formation and development of the curd are primarily categorized into shoot apical meristem (SAM) and three curd development stages, 1) formation stage (FS), where SAM differentiates to the inflorescence meristem (IM). 2) expansion stage (ES), the generation of secondary IMs (each secondary IM becomes a second-order SAM and starts differentiating into new secondary IMs, with the curd diameter increases rapidly). 3) maturation stage (MS), where the IM resumes the ability to differentiate into FM and continues flower development (Sadik, 1962, 1968; Kieffer et al., 1998). Compared with the reproductive development process of other *Brassica* species, such as cabbage, oilseed rape and radish, the differentiation and development of broccoli and cauliflower curds is a flowering reversal phenomenon caused by abnormal differentiation of terminal buds (Zhao et al., 2020a). The apical meristem temporarily loses the ability to differentiate into FM, but the secondary IM continues to differentiate, and the pedicel becomes thicker and the elongation of internodes is inhibited. The curd in cauliflower is caused by the repeated proliferation of the inflorescence or FM development cessation, which has the characteristics of vegetative and reproductive apices (Anthony et al., 1996). However in broccoli, this differentiation cessation occurs before flowering, resulting in a dense collection of small buds on the curd surface (Fujime and Hirose, 1980).

Early studies attempt to elucidate the genetic control of curd development by identifying and characterizing homologous genes in *Arabidopsis thaliana*. An increase in the expression of *LEAFY* (*LFY*) subsequently suppresses *TERMINAL FLOWER 1* (*TFL1*) expression, which initiates flowering by upregulating *APETALA 1* (*AP1*) and *CAULIFLOWER* (*CAL*) in *Arabidopsis* (Goslin et al., 2017; Serrano-Mislata et al., 2017). Studies indicate that the *cal ap1* double mutant in *Arabidopsis* results in the cauliflower phenotype (Bowman et al., 1993). Subsequent research indicates that *BobAP1* and *BobCAL* are the dominant regulatory factors for the curd development cessation (Goslin et al., 2017). In addition, the genomic comparisons between the cauliflower and cabbage have been identified several structural variants crucial for the unique cauliflower phenotype, implicating genes such as *BobFLC* and *BobFRI* in curd initiation, *BobWUS* and *BobMP* in inflorescence proliferation, *BobCAL*, *BobAP1* and *BobSEP3* in floral development cessation and the potential negative regulatory genes in FM, such as *BobAGL14*, *BobSVP* and *BobCCE1* (Guo et al., 2021). Similarly, a total of 86 quantitative trait loci (QTL) were identified which were associated with the agronomic traits of curds in *Brassica oleracea*, such as germination time, and days from germination to flowering (Lan and Paterson, 2000). Furthermore, a total of 20 QTLs were detected in association with the basal diameter, stalk length, stalk angle, and curd solidity of the cauliflower (Zhao et al., 2020b). Additionally, a high-density genetic map including 2741 SNPs was constructed to uncover the cessation of floral development, which further indicates that the formation and development of curd in cauliflower is subjected to multi-gene regulations having a complex regulatory mechanism (Irwin et al., 2016). However, the cessation of cauliflower curd development occurs earlier compared to broccoli curd development. Therefore, the molecular mechanisms and the regulatory network that leads to the special structure of broccoli curd remain unrevealed.

Plant phytohormones play pivotal regulatory roles in the growth, development, and physiological processes, including the flowering process (Liu et al., 2022; Xu et al., 2022). Auxin (AUX) has a critical role in determining the number and identity of floral organs (Cheng and Zhao, 2007). Research indicates that AtAG regulates *AtCRC* and inhibits the expression of *AtTRN2* to modulate AUX homeostasis, thereby controlling the determinacy of the FM (Yamaguchi et al., 2017). The gene *WUSCHEL* (WUS) responds to AUX to maintain the proliferation and differentiation of apical stem cells in *Arabidopsis* (Mayer et al., 1998). At stage 6 of the flower development, the AG-WUS-KNU module precisely controls the maintenance program of FM stem cells (Lenhard et al., 2001; Payne et al., 2004; Prunet et al., 2008; Kwaniewska et al., 2021). Similarly, the expression of *BobLFY* is also influenced by the maximum concentration of AUX, which marks the initiation sites of FMs (Azpeitia et al., 2021). Several studies offer insights into the potential hormonal influences on the unique meristem formation in broccoli curds. However, the specific molecular mechanisms underpinning broccoli curd formation and development remain largely unexplored. Therefore, the objective of this study was to further understand the formation and development of curds in broccoli. The curds with four different development stages were sampled and subjected to transcriptome sequencing. The genes expression profiles and the regulatory networks and metabolic pathways in which the identified DEGs participate should be analyzed in each development stage.

## 2 Materials and methods

### 2.1 Plant materials

**Figure 1:**
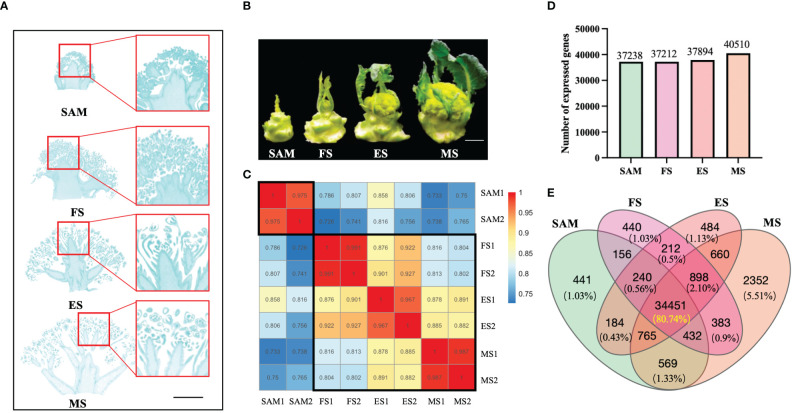
Phenotypes and overall gene expression profiles at four stages of curd development. **(A)** Specific tissues used for constructing RNA libraries at different stages of curd development (shoot apical meristem (SAM), formation stage (FS), expansion stage (ES), maturation stage (MS)), Scale bars=1 cm; **(B)** Phenotypes of broccoli curd formation and development at four stages (SAM, FS, ES, MS), Scale bars=5 cm); **(C)** Samples correlation of four development stages; **(D)** Number of expressed genes at each curd development stage; **(E)** Venn diagram showing the number of commonly or uniquely expressed genes.

The KRJ-012 homozygous broccoli seeds used in this study were provided by associate professor Hanmin Jiang of the Tianjin Kernel Vegetable Research Institute, Tianjin, China. The seeds were cultivated in pots in a greenhouse with a photoperiod of 16 hours light/8 hours dark and temperatures varying between 15°C and 28°C. Thirty days old seedlings were transplanted to the field. Seventy days after transplantation, starting from the appearance of the meristem in the broccoli curd, samples were taken from the apex of the curd every seven days (**Figure 1A**). The stages included: (1) SAM (0 days); (2) FS (7 days); (3) ES (14 days); (4) MS (21 days). The samples from two biological replicates were immediately stored in liquid nitrogen and later kept at -80°C for subsequent RNA extraction and sequencing analysis.

### 2.2 Paraffin embedding, sectioning, and staining

Fresh tissues were immediately fixed using a fixative solution (70% (v/v) ethanol, 5% (v/v) formalin and 5% (v/v) glacial acetic acid) for 48 hours. The trimmed tissues were then dehydrated using a gradient alcohol and embedded in melted paraffin wax. After solidification, the paraffin slicer were sliced with a thickness of 4 μm. The tissue is flattened when the slice floats on the 40 °C warm water of the spreading machine, and then picked up by the glass slides and baked in the oven at 60 °C. The tissue sections were stained with safranin O staining solution and plant solid green staining solution, then scanned and imaged using the Pannoramic MIDI scanner (3DHISTECH, Hungary).

### 2.3 RNA library construction and high-throughput sequencing

Total RNA of the samples was extracted using the RNeasy Kit (Qiagen, China), followed by mRNA enrichment using oligo (dT). The enriched mRNA was subjected to fragmentation, reverse transcription, and PCR amplification for RNA-seq libraries preparation. RNA-seq libraries in this study were sequenced on the BGISEQ-500 platform (Beijing Genomics Institute, China). The analyses were conducted with two biological replicates.

### 2.4 Transcriptome data analysis

The raw sequencing reads were subjected to filtering using SOAPnuke software (https://github.com/BGI-flexlab/SOAPnuke) with the following criteria: 1) removal of reads containing adapters (adapter contamination); 2) removal of reads with unknown base content exceeding 5%; 3) removal of low-quality reads (reads with a proportion of bases with quality values less than 15 and greater than 20% of the total bases were considered low-quality reads). For each sample, a minimum of 4 Gb of raw data, including over 50 million clean reads were required to obtain sufficient transcriptional information. Clean reads were aligned and classified against the reference genome and transcript sequences of *Brassica oleracea* var. *oleracea* (https://www.ncbi.nlm.nih.gov/datasets/genome/GCF_000695525.1/, Parkin et al., 2014) using Bowtie2 (v2.3.4.3) (http://bowtie-bio.sourceforge.net/Bowtie2/index.shtml). Transcript abundances were calculated using StringTie software (Shumate et al., 2022), and normalization was performed using the DESeq2 package in *R* software (Love et al., 2014).

### 2.5 Identification of DEGs

RSEM (v1.3.1) (http://deweylab.biostat.wisc.edu/rsem/rsem-calculate-expression.html) was used for gene expression quantification, |log_2_(A/B)| >1 and q value <0.01 were used as standards to identify DEGs, where A and B represent FPKM value of two groups respectively.

### 2.6 GO and KEGG and genomes analysis of DEGs

GO analysis of DEGs was performed using the agriGO platform (http://bioinfo.cau.edu.cn/agriGO/), with a hypergeometric test was performed to identify significantly enriched GO terms (corrected p value <0.05). To further visualize statistically significant overexpressed GO terms, GO and KEGG enrichment analysis of DEGs was performed using the enrichGO and enrichKEGG functions in the clusterProfiler (Wu et al., 2021).

### 2.7 Gene expression pattern analysis of DEGs

Pearson correlation coefficient and hierarchical clustering for all samples were analyzed using the “cor” and “hclust” functions in *R* software (version 3.0.1) with default values. Hierarchical clustering and Heatmap cluster analysis of DEGs was performed using the *R* package “gplots” and “pheatmap”, respectively.

### 2.8 Validation of gene expression levels by qRT-PCR

Differential expression patterns of representative genes detected by transcriptome data were validated by qRT-PCR analysis. To detect the corresponding genes, specific primer pairs were designed (**Supplementary Table 6**). The *Bolactin* gene from broccoli was selected as an internal reference. Faststart Universal SYBR Green Master (Roche, Germany) was used in all experiments. Relative expression levels of genes were calculated by the comparative 2^-ΔΔCT^ method based on the manufacturer’s recommendations. Three batches of RNA independently isolated from each sample were used, and three technical replicates were performed to ensure the reliability of the quantitative analysis.

## 3 Results

### 3.1 Overview of transcriptome data

The samples of curds at four different stages were conducted to transcriptome sequencing (**Figures 1A, B**). Results showed that a total of 350,731,464 clean reads were filtered from 371,602,504 raw reads. The alignment rates of these clean reads to the reference genome were 83.64% for SAM, 84.04% for the FS, 84.67% for the ES, and 83.95% for the MS (**Supplementary Table 1**). The correlation analysis of these sequencing samples indicates that the correlation was higher between the samples within a group (*r* > 0.96) (**Figure 1C**). Besides, the FS, ES, and MS groups exhibited higher correlations (*r* > 0.87) compared to the SAM group. These results indicate that the sequencing quality is satisfied and the clean reads are suitable for further analysis. Based on the function annotation of these clean reads detected in each sample, a total of 42,667 genes were identified (**Supplementary Table 2**), with 37,238, 37,212, 37,894, and 40,510 genes showing transcriptional expression at SAM, FS, ES, and MS, respectively (**Figure 1D**). Among these expressed genes, 441 genes showed uniquely expression at the SAM stage. 440, 484 and 2352 genes were exclusively expressed at the FS, ES, and MS, respectively (**Figure 1E**). Furthermore, 34,451 genes, constituting 80.74% of the total detected genes, were commonly expressed at all four stages of curd development (**Figure 1E**).

### 3.2 DEGs between SAM and three stages of curd development were identified

**Figure 2:**
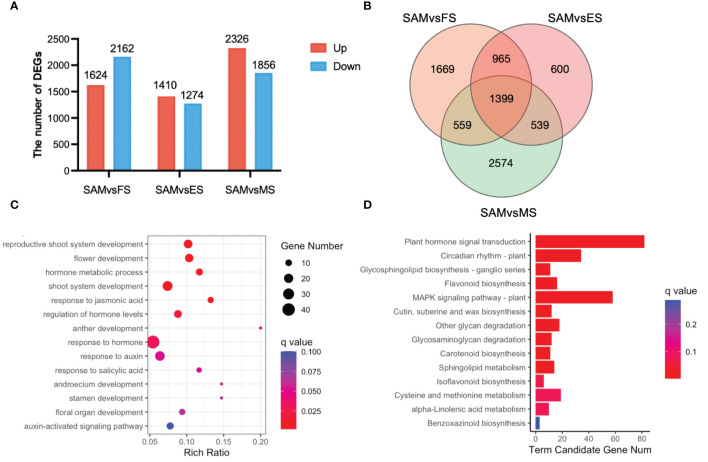
DEGs identified in three pairwise comparisons (SAM vs. FS, SAM vs. ES, and SAM vs. MS). **(A)** The number of DEGs between each two groups (red represents upregulation, blue represents downregulation); **(B)** Venn diagram of DEGs; **(C)** GO functional enrichment of DEGs between SAM and the curd development stages (FS, ES, MS); **(D)** KEGG enrichment of DEGs between the SAM and the curd development stages (FS, ES, MS). DEGs were identified based on the criteria of |log_2_(fold change)| >1 and q value < 0.01.

To investigate candidate genes functioning in the transition from SAM to curd formation, the transcriptional expression levels of genes were compared between SAM and three curd development stages, FS, ES, and MS, respectively. A total of 10,652 genes exhibited differential expression levels. Specifically, in the comparison between SAM and FS, there were 1,624 DEGs upregulated and 2,162 DEGs downregulated. In the SAM vs. ES comparison, 1,410 DEGs were upregulated and 1,274 were downregulated. In the SAM vs. MS comparison, 2,326 DEGs were upregulated and 1,856 were downregulated. (**Figure 2A**). Among them, 965 genes displayed significantly differential expression levels in SAM vs. FS and SAM vs. ES, 559 genes displayed significantly differential expression levels in SAM vs. FS and SAM vs. MS, and 965 genes displayed significantly differential expression levels in SAM vs. FS and SAM vs. ES (**Figure 2B**). A total of 1,399 genes showed significantly differential expression patterns at SAM stage compared with each of the three curd development stages (**Figure 2B**; **Supplementary Table 3**).

These overlapping DEGs between the SAM and three curd development stages likely play crucial role in regulating curd formation and development. Subsequently, the possible genetic regulatory networks and pathways enriched by these 1,399 overlapping DEGs were explored by GO and KEGG analysis. The GO enrichment assay indicates that among the GO terms targeted by these overlapping DEGs, the reproductive-associated terms, such as reproductive shoot system development (GO:0090567), flower development (GO:0009908), anther development (GO:0048653), stamen development (GO:0048443), and floral organ development (GO:0048437); phytohormones, especially AUX, JA and SA-associated terms, such as hormone metabolic processes (GO:0042445), response to jasmonic acid (GO:0009753), regulation of hormone levels (GO:0010817) and response to AUX (GO:0009733), were significantly enriched. Moreover, KEGG pathway assay also indicates that plant hormone signal transduction (ko04075) and circadian rhythm (ko04712) were significantly enriched by these overlapping DEGs, suggesting their pivotal roles in the complex regulation of the transition from SAM to curd formation.

In addition, DEGs in temporal stages specific manner (SAM vs. FS, FS vs. ES, and ES vs. MS) were identified. GO term enrichment analysis revealed that DEGs in SAM vs. FS were predominantly involved in auxin-activated signaling pathway (GO:0009734), photosystem I (GO:0009522) and shoot system development (GO:0048367) (**Supplementary Figure 3**). KEGG pathway analysis revealed that DEGs in SAM vs. FS were predominantly annotated in the plant hormone signal transduction (ko04075) and circadian rhythm (ko04712) pathways (**Supplementary Figure 3**), which are consistent across the SAM and the later three developmental stages. However, during the transition from SAM to FS, the pentose phosphate pathway (ko00030) was also identified, indicating that this pathway may also be involved in the curd formation. In the FS vs. ES comparison, significant annotation of photosystem (GO:0009521) in the GO terms and photosynthesis-antenna proteins (ko00196) in the KEGG pathway suggests the crucial role of photosynthesis in the transition from curd formation to enlargement. In the ES vs. MS comparisons, DEGs were significantly annotated in floral organ development-related GO categories, including pollen wall assembly (GO:0010208), flower development (GO:0009908) and anther development (GO:0048653). Moreover, DEGs in the ES vs. MS comparisons were predominantly annotated in the starch and sucrose metabolism (ko00500) KEGG pathway (**Supplementary Figure 3**). These analysis results were consistent with the following comparison results of the three stages of curd development.

### 3.3 DEGs associated with AUX, GA and ABA signal pathways were highly expressed at SAM stage

**Figure 3:**
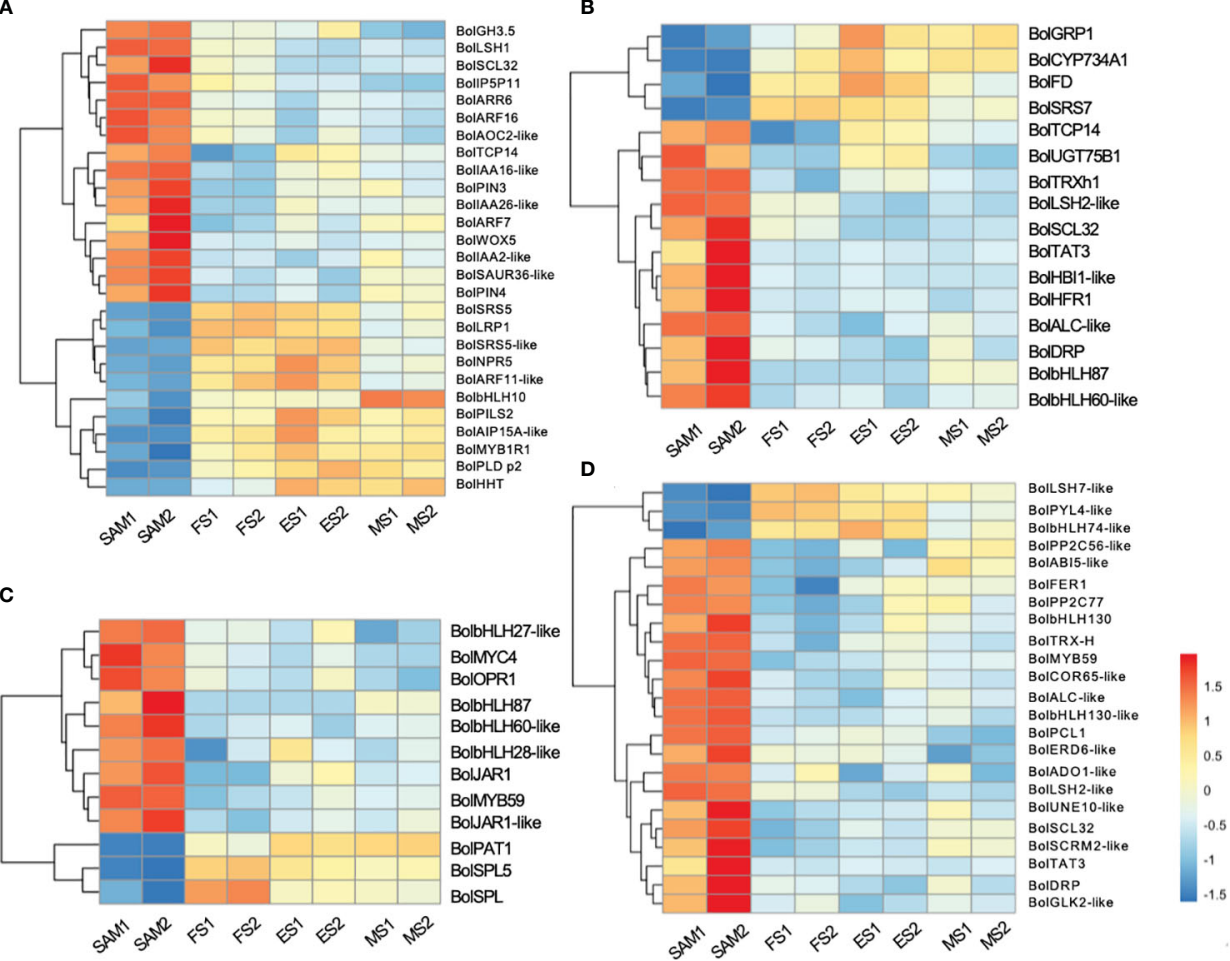
Transcriptional expression profiles of DEGs involved in the phytohormone signal transduction pathways. The DEGs were identified in three pairwise comparisons (SAM vs. FS, SAM vs. ES, and SAM vs. MS). **(A)** DEGs related to AUX; **(B)** DEGs related to GA; **(C)** DEGs related to JA; **(D)** DEGs related to ABA. DEGs were identified based on the criteria of |log_2_(fold change)| >1 and q value < 0.01.

GO term annotation and KEGG pathway enrichment analyses revealed that phytohormones may play a key regulatory role in the formation of curd (**Figures 2C, D**). Among the DEGs involved in phytohormone signal transduction pathways, 27 genes were significantly enriched in AUX signal pathway (**Figure 3A**), 18 in the GA signal pathway (**Figure 3B**), 12 in the JA signal pathway (**Figure 3C**), and 23 in the ABA signal pathway (**Figure 3D**).

**Figure 4:**
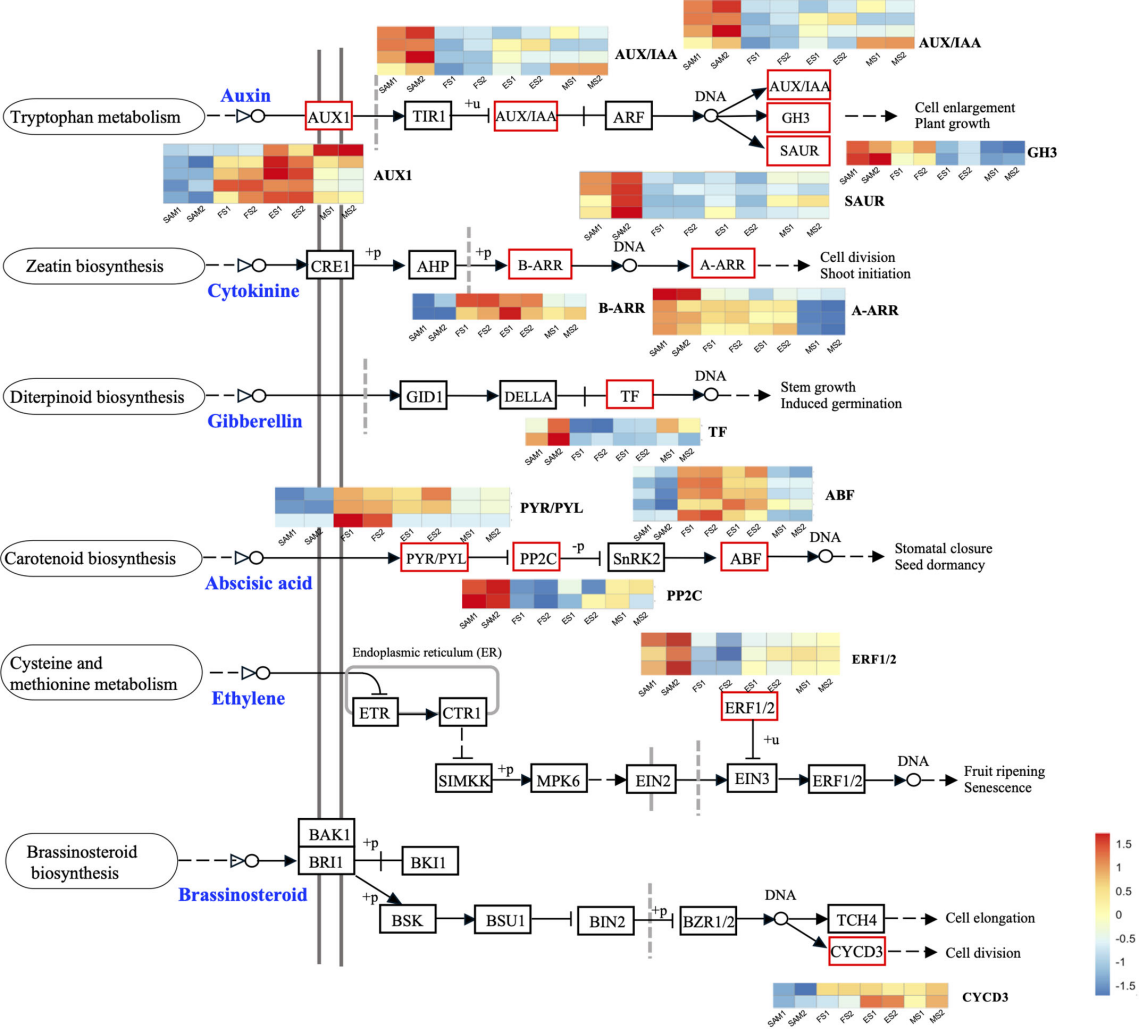
Plant hormone signal transduction pathway components and the expression profiles of DEGs. The DEGs were identified in three pairwise comparisons (SAM vs. FS, SAM vs. ES, and SAM vs. MS), based on the criteria of |log_2_(fold change)| >1 and q value < 0.01.

AUX, GA, and ABA are known for their roles in organ identity and development (Wei et al., 2021). Gene expression profile assay indicates that genes involved in AUX signal pathways, such as *BolAUX/IAA*, *BolGH3*, *BolSAUR*, *BolARF16*, *BolARF7*, *BolARR6*, *BolIAA16-like*, *BolSAUR36-like* and *BolWOX5*, were highly expressed at the SAM stage, and then downregulated at the subsequent stages of curd development (**Figures 3A**, **4**). Notably, the AUX transduction negative regulator *BolNPR5* was upregulated at FS, ES and MS compared to SAM (**Figure 3A**).

GA plays a crucial role in regulating the proliferation and differentiation of the SAM and the formation of buds and floral buds. Among the 22 DEGs related to the GA signal pathway, the majority of genes were highly expressed at SAM stage, but showed lower expressions during the subsequent development stages, including four negative regulators: *BolUGT75B1*, *BolTAT3*, *BolGLK2-like*, and *BolLDSH2-like*. Notably, the expression of *Gibberellin-Regulated Protein 1* (*BolGRP1*) was low at SAM stage but upregulated at the development stages (**Figure 3B**). In the ABA signal pathway, ABA negative regulatory protein coding genes *BolABI5-like*, *BolPP2C56-like* and *BolPP2C77* were highly expressed at SAM stage, but significantly downregulated at the development stages, while the positive regulator *BolPYL4* was upregulated at FS, ES and MS compared to SAM (**Figure 3D**). The upregulation of *BolPYL4* may directly affect the transcription levels of downstream gene *BolABF*, thereby positively regulating the differentiation process of SAM (**Figure 4**).

Among the 12 DEGs related to the JA signal pathway, 9 genes were highly expressed at SAM stage and significantly downregulated during the subsequent development stages, including the JA signaling activator *BolJAR1*, *BolbHLH27-like*, *BolMYC4*, *BolMYB59*, *BolTAT3* and *BolbHLH87*. Notably, the negative regulator *BolSPL5* and *BolSPL10* were highly expressed at FS, ES and MS (**Figure 3C**), suggesting that genes related to JA negatively regulate the development of SAM, promoting the transition from SAM to IM. In the cytokinin (CTK) pathway, *BolA-ARR*, which positively regulates cell division, was highly expressed at SAM, FS and ES, but its expression decreased at MS (**Figure 4**).

### 3.4 DEGs related to shoot apical and flower development were identified in comparison between SAM and curd development stages

**Figure 5:**
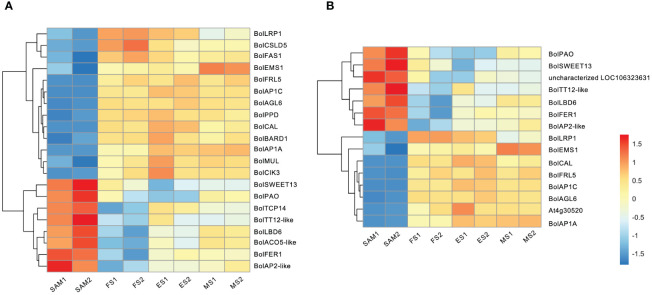
Transcriptional expression profiles of DEGs identified in three pairwise comparisons (SAM vs. FS, SAM vs. ES, and SAM vs. MS). **(A)** DEGs involved in reproductive shoot system development; **(B)** DEGs involved in flower development. The DEGs were identified based on the criteria of |log_2_(fold change)| >1 and q value < 0.01.

The formation of curds is a flowering reversal phenomenon caused by abnormal differentiation of terminal buds. During the FS, the apical IM temporarily loses its capacity to differentiate into floral organs but continues to differentiate into secondary IMs. In this study, reproductive shoot system development took the first place in GO terms in the SAM vs. FS, SAM vs. ES, and SAM vs. MS (**Figure 2C**). Therefore, it is speculated that genes involved in the reproductive shoot system development may play a crucial role in the floral development cessations, and significantly influence the unique structure of the curd. Among the 22 genes associated with the reproductive shoot system development (**Figure 5A**), 13 of them exhibited low expression levels at the SAM stage but were significantly upregulated at the development stages (FS, ES, MS), such as *BolLRP1*, *BolFAS1*, *BolEMS1*, *BolFRI*, *BolAP1A*, *BolAP1C*, *BolAGL6*, and *BolCAL*. On the contrary, 9 genes were highly expressed at the SAM stage, such as sucrose transporter *BolSWEET13*, *BolTT12-like*, *BolTCP14*, and *BolAP2-like*. Additionally, a part of the DEGs associated with the reproductive shoot system development were also involved in the flower development process (**Figure 5B**), indicating that these genes are involved in the transformation from IM to FM and the development of floral organs.

### 3.5 Transcription factors with MADS and bHLH domain were extensively involved in the transition of SAM

**Figure 6:**
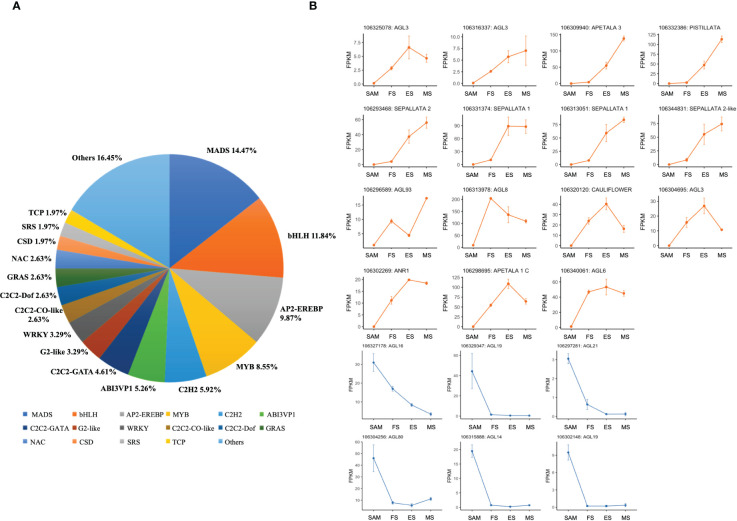
Statistics analysis of transcription factor families enriched in DEGs detected in three pairwise comparisons (SAM vs. FS, SAM vs. ES, and SAM vs. MS). **(A)** Proportion of each transcription factor family; **(B)** Transcriptional expression profiles of transcription factor with MADS domain at SAM stage and three development stages of curd (orange and blue line respectively represents upregulation and downregulation during the curd development stages compared to SAM).

Following the functional enrichment and pathway analysis of DEGs from overlapping genes in three pairwise comparisons (FS vs. SAM, ES vs. SAM, MS vs. SAM), it became evident that multiple transcription factors (TFs) likely play a pivotal role at the SAM stage. Given the significant role of TFs in regulating plant morphology, growth, and development (Wang et al., 2019; Jiang et al., 2022), the TF families among the DEGs shared in SAM vs. FS, SAM vs. ES, and SAM vs. MS were classified. In this comparison, 152 DEGs were identified, belonging to 31 TF families. The top six families, based on percentage representation, included MADS (14.47%), bHLH (11.84%), AP2-EREBP (9.87%), MYB (8.55%), C2H2 (5.92%), and ABI3VP1 (5.26%) (**Figure 6A**). Notably, the MADS-box gene family, a large and crucial group in plants, especially for flower development, was prominently featured. Within this family, 6 DEGs, such as *BolAGL16*, *BolAGL19*, *BolAGL21*, were observed to be downregulated (**Figure 6B**), while the majority of the DEGs belonging to the MADS family were significantly upregulated during curd development (**Figure 6B**), such as *BolAGL3*, *BolAP1-A*, *BolAP3*, *BolPISTILLATA*, *BolSEP2* and *BolSEP1*. This pattern indicates that the MADS-box TF family plays a critical role in the transition from SAM to IMs.

### 3.6 DEGs associated with photosynthesis were highly expressed at the ES and MS

**Figure 7:**
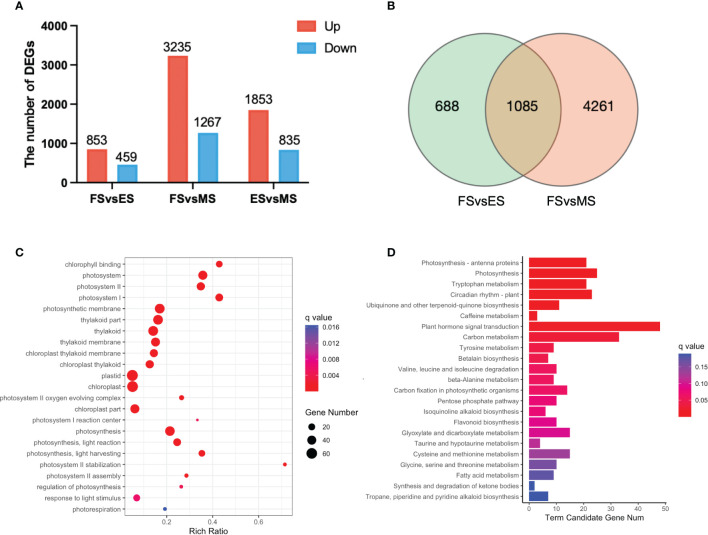
DEGs between the three stages of curd development (ES, FS, MS). **(A)** The number of upregulated and downregulated DEGs between each group; **(B)** Venn diagram of DEGs between the two groups (FS vs. ES, FS vs. MS); **(C)** GO term enrichment of overlapping DEGs in two pairwise comparisons (FS vs. ES, FS vs. MS); **(D)** KEGG enrichment of overlapping DEGs in two pairwise comparisons (FS vs. ES, FS vs. MS). DEGs were identified based on the criteria of |log_2_ (fold change)| >1 and q value < 0.01.

The overlapping DEGs in the FS vs. ES, FS vs. MS and ES vs. MS were analyzed to investigate the flowering reversal phenomenon in broccoli curds (**Supplementary Table 4**). The results showed that there were 853 upregulated and 459 downregulated genes in FS vs. ES, 3,235 upregulated and 1,267 downregulated genes in FS vs. MS, 1,853 upregulated and 835 downregulated genes in ES vs. MS (**Figure 7A**). Additionally, 1,085 DEGs shared in the FS vs. ES and FS vs. MS were identified (**Figure 7B**).

**Figure 8:**
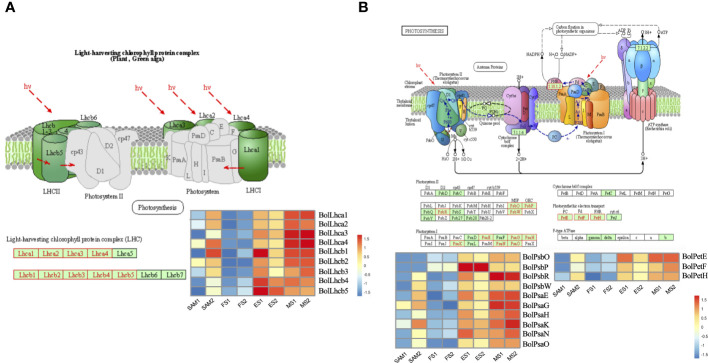
Transcriptional expression profile of genes involved in photosynthesis. **(A)** Transcriptional expression profile of genes which match Photosynthesis-antenna protein pathway (ko00196). These genes showed significant expression levels in in two pairwise comparisons (FS vs. ES, FS vs. MS). **(B)** Transcriptional expression profile of genes which match Photosynthesis pathway (ko00195). These genes showed significant expression levels in two pairwise comparisons (FS vs. ES, FS vs. MS). DEGs were identified based on the criteria of |log_2_(fold change)| >1 and q value < 0.01.

Twenty-three significantly enriched GO terms and KEGG pathways were identified based on the DEGs shared in FS vs. ES and FS vs. MS (**Figure 7**). Specifically, GO term enrichment was linked to photosystems (I, II), photosynthesis membrane, thylakoid part, thylakoid membranes and chloroplasts (**Figure 7C**). The transcriptional expression level analysis of DEGs involved in the photosystem (GO:0009521) revealed that 33 photosynthesis-related genes were significantly upregulated at FS and ES (**Supplementary Figure 1**), which may contribute to the formation of the FM. Additionally, carbon metabolism, amino acid metabolism and signal transduction pathways were enriched in both pairwise comparisons (FS vs. ES and FS vs. MS). The detailed metabolic pathways included photosynthesis-antenna protein (ko00196), photosynthesis (ko00195), tryptophan metabolism (ko00380), and plant cycle rhythm (ko04712) (**Figure 7D**). DEGs related to photosynthesis-antenna protein (ko00196) and photosynthesis (ko00195) were also highly expressed at ES and MS (**Figure 8**). The joint analysis of GO and KEGG revealed that photosynthesis-related DEGs, such as *BolLhca1*, *BolLhcb1* and *BolPsbO*, play a pivotal regulatory role in curd expansion and maturation.

### 3.7 A series of genes related to flower and pollen development were highly expressed at MS

**Figure 9:**
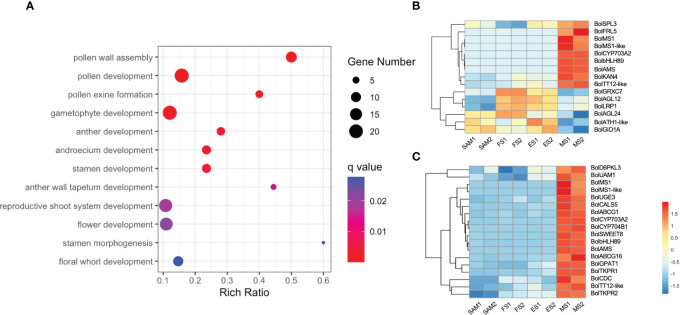
Analysis of DEGs identified in three pairwise comparisons (MS vs. SAM, MS vs. FS, and MS vs. ES). **(A)** GO term enrichment related to flower development; **(B)** Expression profile of DEGs involved in flower development (GO:0009908); **(C)** Expression profile of DEGs involved in pollen development (GO:0009555). DEGs were identified based on the criteria of |log_2_ (fold change)| >1 and q value < 0.01.

A total of 2,170 overlapping DEGs were identified in the comparison of MS vs. SAM, MS vs. ES and MS vs. FS (**Supplementary Figure 2**, **Supplementary Table 5**). GO term enrichment analysis revealed that these DEGs predominantly involved in biological processes such as pollen development (GO:0009555), anther development (GO:0048653), and flower development (GO:0009908) (**Figure 9A**). This finding reveals that at the curd development stages, the IM regains the capacity to differentiate FM and resume the flower development process, involving the formation of floral organs like pollen tubes, anthers, and stamens. Specifically, the expression profile of genes related to flower development shows that the expression level of *BolFRI-like5*, *BolKAN4*, *BolbHLH89* and *BolTT12-like* were significantly upregulated at MS (**Figure 9B**), indicating their vital role in flower organ formation. Furthermore, a consistent pattern of significant upregulation at the MS was observed among 18 DEGs related to pollen development (**Figure 9C**). Additionally, two AG-like family genes, *BolAGL12* and *BolAGL24* were observed, exhibited a high expression level at ES and FS, but significantly decreased at MS. Therefore, it is speculated that *BolAGL12* and *BolAGL24* may negatively regulate the differentiation process of FM in broccoli curd. Similarly, *BolLRP1* exhibited a high expression level at ES and FS, followed by a significant reduction at MS, suggesting its positive influence on the development of lateral IMs in the broccoli curds.

### 3.8 Genes involved in starch and sucrose metabolic pathways accumulate highly at the transcriptional level at MS

**Figure 10:**
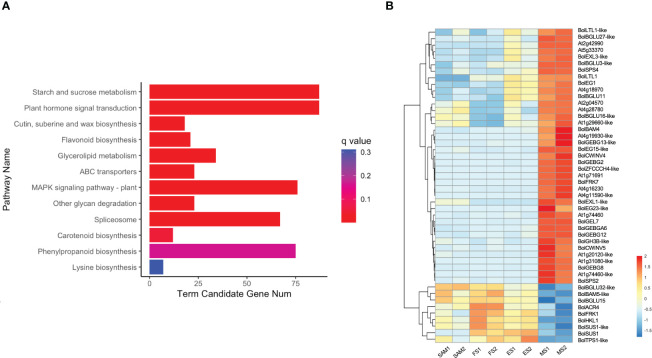
Analysis of DEGs identified in three pairwise comparisons (MS vs. SAM, MS vs. FS, and MS vs. ES). **(A)** KEGG enrichment analysis of DEGs; **(B)** Transcriptional expression profile of DEGs involved in starch and sucrose metabolism (ko00500). DEGs were identified based on the criteria of |log_2_ (fold change)| >1 and q value < 0.01.

KEGG pathway enrichment analysis revealed that 87 overlapping DEGs in MS vs. SAM, MS vs. FS and MS vs. ES were predominantly annotated in the starch and sucrose metabolism (ko00500) pathway and the plant hormone signal transduction (ko04075) pathway (**Figure 10A**), suggesting a potential connection of these pathways with the regulation of the curd maturation. The expression profile of genes involved in the starch and sucrose metabolism pathway showed that 39 genes, including 16 from the GELP gene family, maintained low expression levels at the SAM stage, ES and FS, but significantly increased at the MS (**Figure 10B**). These processes are essential for sustaining the cellular energy supply and the growth and development of organisms.

### 3.9 Validation of DEGs expression patterns by qRT-PCR

**Figure 11:**
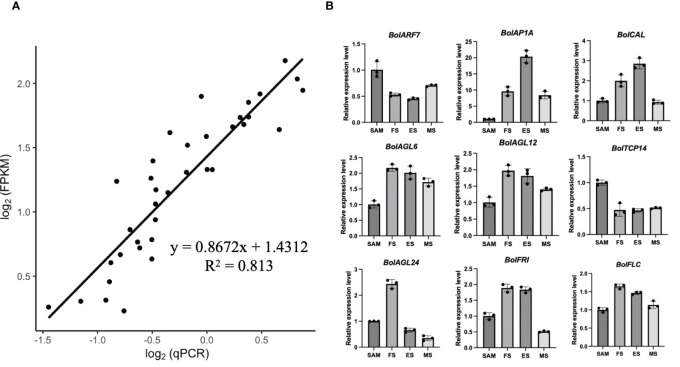
Validation of transcriptional expression levels of the representative DEGs by qRT-PCR. **(A)** The correlation between transcriptional data and qRT-PCR mean values for the nine DEGs; **(B)** qRT-PCR analysis of the representative DEGs (Bar graphs with error bars depict the mean and standard deviation values).

In order to validate the RNA-seq quality, quantitative reverse-transcription PCR (qRT-PCR) analysis was performed on the 9 representative DEGs identified in the SAM and curd development stages, including *BolARF7*, *BolAP1A*, *BolCAL*, *BolAGL6*, *BolAGL12*, *BolTCP14*, *BolAGL24*, *BolFRI* and *BolFLC*. The selection of these genes was based on their prominent roles in the development processes as identified in the RNA-seq data. The results of the qRT-PCR analysis showed a high degree of consistency with the expression patterns obtained from the high-throughput sequencing data (**Figure 11**). This consistency reinforced the reliability of the RNA-seq in capturing the gene expression dynamics across the various stages of the curd development.

## 4 Discussion

### 4.1 Endogenous hormones are closely related to the proliferation and differentiation of the SAM

**Figure 12:**
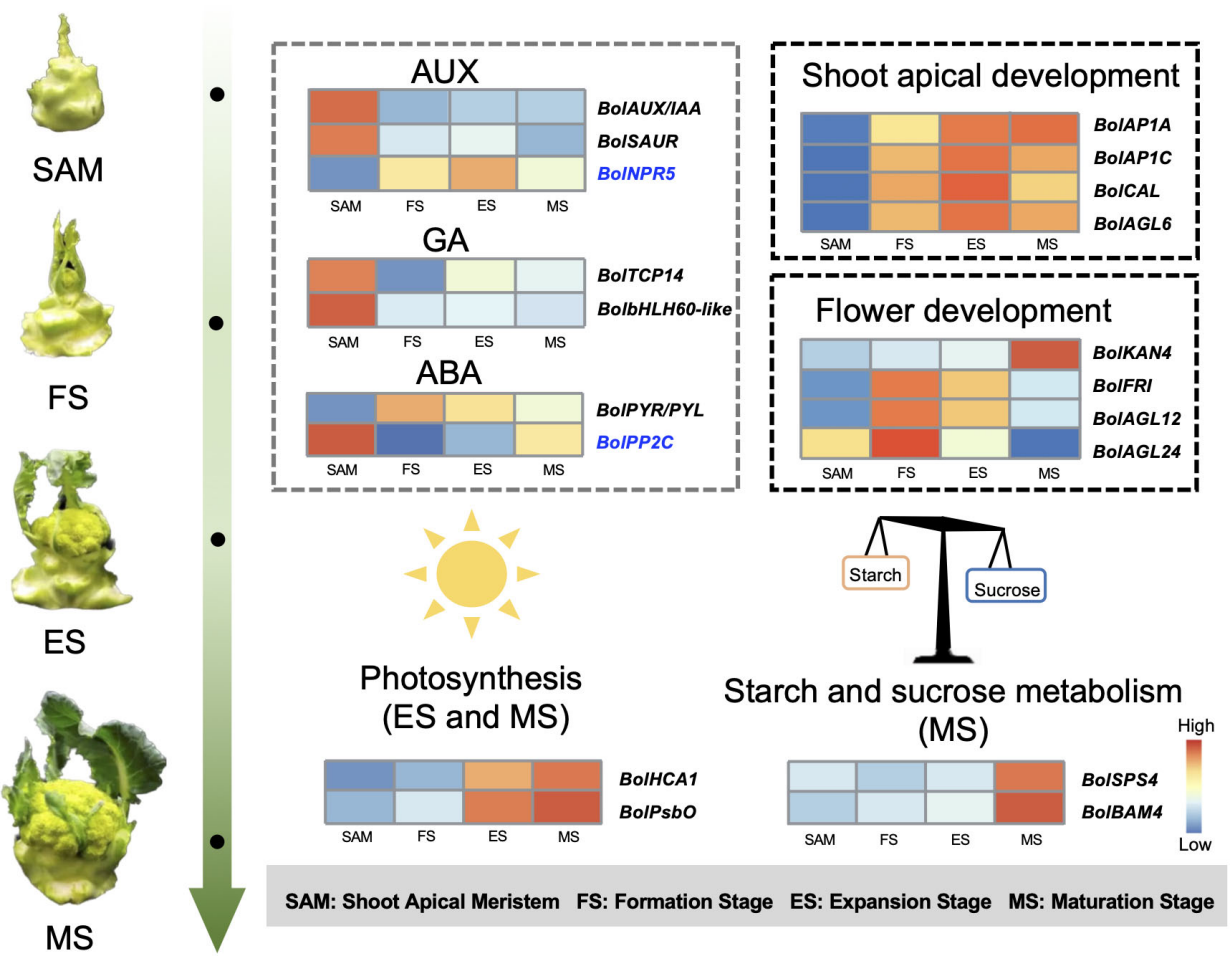
Pattern diagram of key regulatory factors at SAM stage and curd development stages (The genes marked in blue represent negative regulatory factors related to specific hormones.).

The role of endogenous hormones in the floral transition has been extensively studied in model plants (Davis, 2009), but their function in the formation of curd in broccoli remains largely unknown. This study proposes that AUX, GA, ABA signaling pathways are involved in regulating the formation and development of broccoli curd, forming a complex and interconnected hormone signaling network to coordinate the proliferation and differentiation of meristem at different stages (**Figures 3**, **4**). It has been reported that AUX gradually accumulates in the SAM during the flowering transition in strawberries, indicating a crucial role of AUX in flowering transition (Hou and Huang, 2005). *AUX/IAA* and *SAUR*, play crucial roles in AUX signaling, affecting root, shoot and flower development (Guilfoyle, 2015; Luo et al., 2018). AUX/IAA always act as transcriptional repressors (Cheng et al., 2021), inhibiting *BolARF* activity and the transcription of AUX-responsive genes by competing with ARF proteins to bind to the SCFTIR1/AFB complex (Figueiredo and Strader, 2022). The high expression of *BolAUX/IAA* in SAM may be one of the main reasons for the abnormal differentiation of the SAM of the curd (**Figure 12**). Conversely, *BolNPR5*, a negative regulator of the AUX signal transduction pathway, upregulated at the development stage (**Figure 12**), appears to fine-tune the AUX signal transduction, balancing growth-promoting and inhibitory processes (Wang et al., 2015).

GA is known to regulate the SAM proliferation and differentiation (Song et al., 2022). GA-regulated class I TCP-DELLA interactions regulate the development of inflorescence stem apex (Davière et al., 2014). Low expression of *BolTCP14* at curd development stages suggests the accumulation of DELLA proteins, which could be a primary reason for inhibiting the elongation of inflorescence stems and internodes (**Figure 3C**). bHLH60 could interact with DELLA and positively regulate GA-mediated flowering by upregulating *FT* in *Arabidopsis* (Li et al., 2017). The sustained low expression of the *BolbHLH60* during the three stages of curd development may be one of the main reasons for the abnormal differentiation of the apical meristem. In the ABA signal pathway, ABA negative regulatory protein coding gene *BolPP2C* were highly expressed at SAM stage, but significantly downregulated at curd development stages (**Figure 12**), while the positive regulator *BolPYR/PYL* was upregulated (**Figure 12**), which means genes associated with ABA positively regulate the formation and development of the curd. Therefore, the high expression of AUX and GA-related DEGs at the SAM stage may primarily contribute to the regulation of SAM proliferation, the subsequent sustained low expression during development stages may be one of the main reasons for abnormal differentiation of apical meristem. DEGs related to ABA are closely associated with the differentiation of SAM into IM in the curd.

### 4.2 Several genes play key regulatory roles in curd development

In this study, most of the genes related to the reproductive shoot system development and flower development was significantly upregulated at the FS, ES and MS, including key genes like *BolLRP1*, *BolFAS1*, *BolEMS1*, *BolFRI*, *BolAP1A*, *BolAP1C*, *BolCAL* and *BolAGL6* (**Figures 5A, B**). Studies have shown that *BobAP1* and *BobCAL* are the dominant regulators of the floral development cessation of cauliflower (Bowman et al., 1993; Kempin et al., 1995). *BobCAL*-transformed cauliflowers were unable to form curds, but instead produced green, loose inflorescences composed of flower buds (Zhao et al., 2003). Subsequent studies further confirmed that *BobCAL* plays a key regulatory role in the curd formation of cauliflower (Goslin et al., 2017; Azpeitia et al., 2021). In this study, *BolAP1* and *BolCAL* were significantly upregulated at the FS, ES and MS compared to SAM (**Figure 12**), indicating their potential role in the curd formation and expansion. AGL6, along with AP1 and CAL, belongs to the MADS-box family, plays a role in lateral and floral organ development in *Arabidopsis*, interacting with proteins like SEP1, SEP3, SHP2, AP1, FUL, SVP, AGL24, and AGL42 (Koo et al., 2010). However, the function of *BolAGL6* in broccoli curd development is not yet clear. In this study, the high expression of *BolAGL6* during the three stages of curd development suggests that *BolAGL6* may be involved in the proliferation and differentiation processes of the IM in the curd (**Figure 6B**).

Analysis in this study revealed differential expression of genes like *BolFRI*, *BolAGL12*, *BolAGL24*, and *BolLRP1* in the flower development (**Figure 9B**). Among them, *BolFRI* was significantly upregulated at the MS, potentially influencing the expression of *BolFLC* to inhibit flowering. Conversely, *BolAGL12* and *BolAGL24* regulate the development of FM were significantly downregulated at the MS, likely contributing to the developmental cessation of broccoli curds during the formation of immature buds. The synergistic/antagonistic regulatory effects between these DEGs provide crucial insights into the establishment of broccoli curd morphology.

### 4.3 Expression patterns of representative genes in the meristem of the curd

In *Arabidopsis*, the SAM transitions from the juvenile vegetative meristem (JVM) to the adult vegetative meristem (AVM), and then develops into the inflorescence meristem (IM) and the lateral floral meristems (FM) (Kaufmann et al., 2010; Huijser and Schmid, 2011). We found significant differences in the expression patterns of key genes in the meristems at different stages of broccoli compared to *Arabidopsis*. For instance, *AtAP2L* shows upregulation during the transition from IM to FM in *Arabidopsis* (Chávez-Hernández et al., 2022), whereas its expression starts to decline at the FS in broccoli (**Supplementary Figure 4**). Conversely, *AtTFL1* is significantly downregulated at the FM stage in *Arabidopsis*, but the expression of *BolTFL1* in broccoli begins to increase significantly when the curd begins to form, possibly contributing to the abnormal IM differentiation (**Supplementary Figure 4**). Additionally, *AtAGL24* and *AtFLC* do not show significant changes at the FM stage compared to the IM stage in *Arabidopsis*, but *BolAGL24* and *BolFLC* start to show significant upregulation at FS (**Supplementary Figure 4**), possibly supporting the dense formation of small flower buds in broccoli. In addition, we analyzed the expression patterns of some homolog-specific genes, and found that there were some small changes in the expression patterns of homologous genes, but the overall expression patterns were similar (**Supplementary Figure 5**).

### 4.4 Photosynthesis and Starch and sucrose metabolism facilitating the enlargement and maturation of the curd

Photosynthesis, a crucial chemical reaction in plants, enables the conversion of carbon dioxide and water into energy-rich organic compounds and oxygen through the utilization of light energy by chloroplasts (Kaiser et al., 2019). At the FS, the inflorescence apical meristem of the curd temporarily loses the ability to differentiate into floral organs, but continues to differentiate into secondary IMs. During the subsequent development of the curd, the IM regains the ability to differentiate into FMs. The ongoing photosynthetic activity during this period accumulates energy necessary for the continuous differentiation of floral organs, contributing to the curd’s enlargement.

From the curd enlargement stage, photosynthesis emerges as a key process, capturing sunlight energy and converting it into chemical energy, and then through the action of a series of enzymes, excess glucose is converted into starch and stored in chloroplasts and plastids (Lebon et al., 2005; Ning et al., 2020). At night or when photosynthesis is limited, plants break down starch back into glucose and then further synthesize into sucrose to meet their energy and carbon needs (Delatte et al., 2006). The activity and expression level of *BolSPS4* and *BolBAM4* are closely related to the relative ratio of starch and sucrose. Increased activity of SPS4 enzyme may lead to higher sucrose accumulation and lower starch accumulation (Li et al., 2018), and BAM4 is a starch-degrading factor, *bam4* mutants display a starch-excess phenotype (David et al., 2022). Notably, the expression of *BolSPS4* and *BolBAM4* were significantly higher at the MS compared to earlier stages (**Figure 10B**), suggesting that broccoli may shift towards decomposing starch into small carbon metabolites to provide energy. It is speculated that in the curd development stage, starch and sucrose metabolism supplies energy for the differentiation of FMs, thus promoting curd maturation. This interplay of photosynthesis and carbohydrate metabolism underscores their pivotal roles in the complex process of broccoli curd development.

## 5 Conclusion

This study obtained 350,731,464 clean reads through transcriptome sequencing of broccoli curd at different stages. It is suggested that the roles of *BolAP1A*, *BolAP1C*, *BolCAL*, and *BolAGL6* in the abnormal differentiation of SAM, as well as the involvement of *BolKAN4*, *BolFRI, BolAGL12*, and *BolAGL24* in the development of FM in the broccoli curd. Additionally, the study emphasizes the importance of phytohormones such as AUX, GA, and ABA in SAM proliferation and differentiation, as well as the significant roles of photosynthesis and starch and sucrose metabolism related genes in curd expansion and maturation (**Figure 12**). These insights significantly contribute to understanding the molecular basis of broccoli curd formation and the unique reproductive development of cruciferous plants.

## Data availability statement

The data presented in the study are deposited in the NCBI repository, accession number PRJNA1087260.

## Author contributions

YZ: Conceptualization, Writing – original draft, Data curation. CL: Software, Writing – review & editing. MZ: Methodology, Writing – review & editing. YD: Investigation, Writing – review & editing. JX: Investigation, Methodology, Writing – review & editing. CW: Conceptualization, Funding acquisition, Resources, Supervision, Writing – review & editing.
