# Supplementary material for: Transcriptome profiling reveals key regulatory factors and metabolic pathways associated with curd formation and development in broccoli
Source: Front Plant Sci. 2024 Jul 12;15:1418319. doi: 10.3389/fpls.2024.1418319 (PMC11273133; doi:10.3389/fpls.2024.1418319)
Supplement: Supplementary file 2 [file DataSheet_2.pdf]

Supplementary Material

Transcriptome profiling reveals key regulatory factors and metabolic pathways associated with curd formation and development in broccoli

Yinxia Zhu[1], Ce Liu[2 3], Mengyao Zhao[1], Yuxuan Duan[1], Jingjing Xie[1], Chunguo Wang[1*]
[1]College of Life Sciences, Nankai University, Tianjin 300071, China
[2]Cucumber Research Institute, Tianjin Academy of Agricultural Sciences, Tianjin 300192, China
[3]State Key Laboratory of Vegetable Biobreeding, Tianjin 300192, China
***Correspondence author:** Chunguo Wang, E-mail: wangcg@nankai.edu.cn;

1 Supplementary Figures

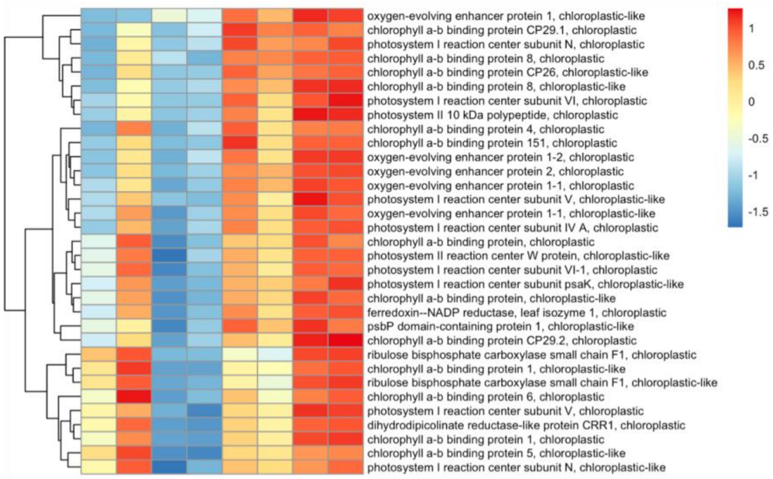

Fig. S1 The transcriptional expression profile of DEGs identified in both pairwise comparisons (FS vs. ES and FS vs. MS) involved in the photosystem (GO:0009521) pathway. DEGs were identified based on the criteria of $|\log_2 (\text{fold change})| > 1$ and q value < 0.01.

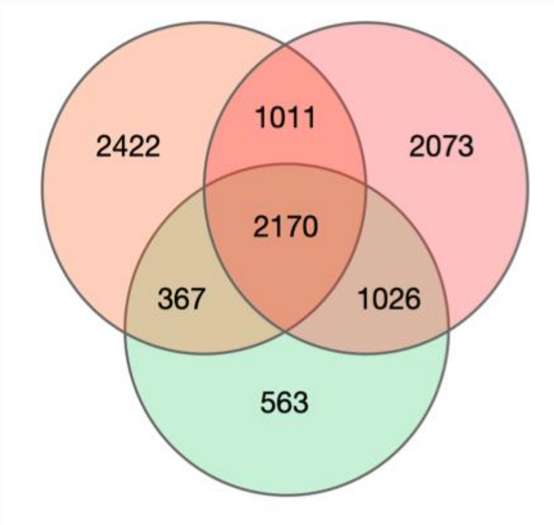

Fig. S2 Venn diagram of overlapping DEGs identified in the comparison of MS vs. SAM, MS vs. ES and MS vs. FS. DEGs were identified based on the criteria of $|\log_2 \text{(fold change)}| > 1$ and q value < 0.01.

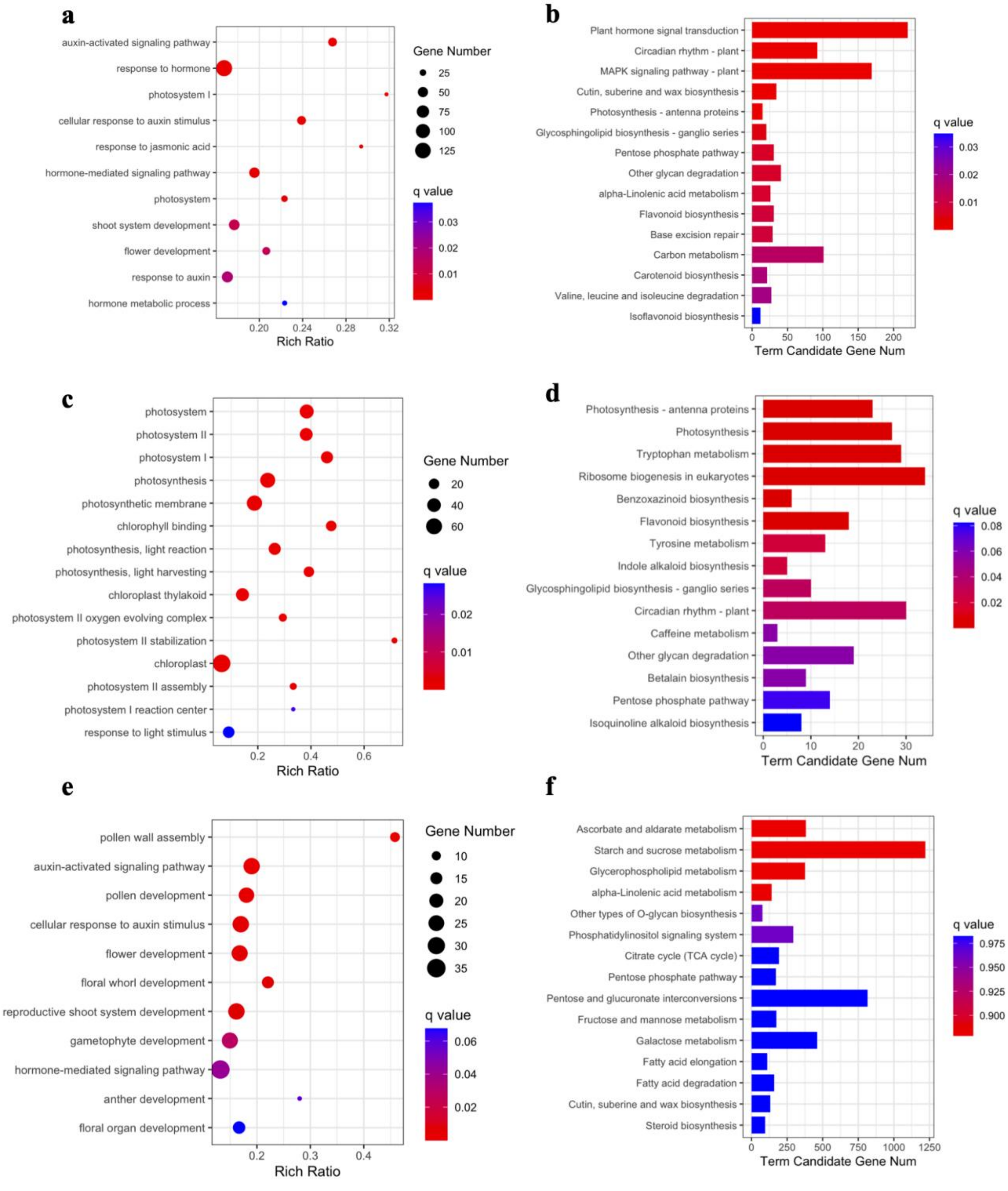

Fig. S3 Analysis of DEGs identified in three pairwise comparisons (SAM vs. FS, FS vs. ES, and ES vs. MS). (a): GO term enrichment analysis of DEGs identified in SAM vs. FS; (b) KEGG enrichment analysis of DEGs identified in SAM vs. FS; (c): GO term enrichment analysis of DEGs identified in FS vs. ES; (d) KEGG enrichment analysis of DEGs identified in FS vs. ES; (e): GO term enrichment analysis of DEGs identified in ES vs. MS; (f) KEGG enrichment analysis of DEGs identified in ES vs. MS. DEGs were identified based on the criteria of $|\log_2 \text{(fold change)}| > 1$ and q value < 0.01.

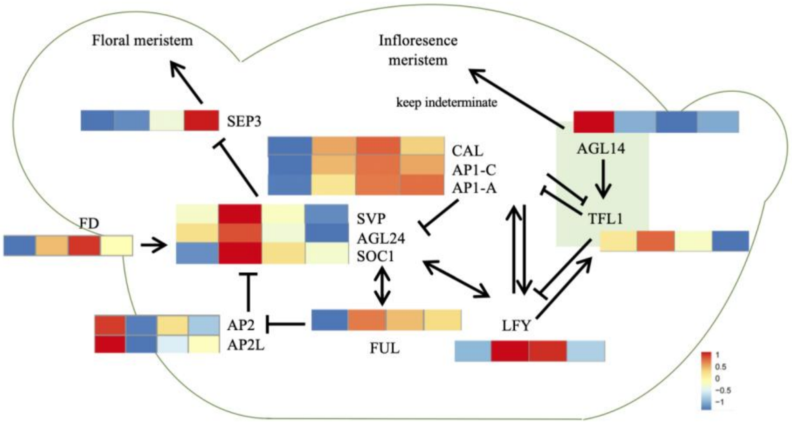

Fig. S4 Regulatory network of key genes in broccoli curd meristem.

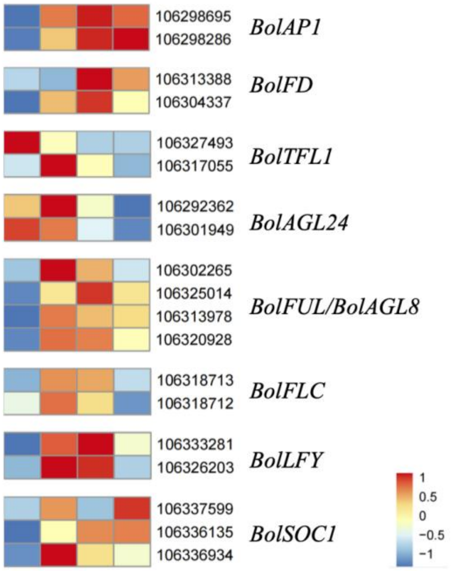

Fig. S5 Expression patterns of key regulatory homologous genes.

2 Supplementary Tables (Zip, 12MB)

Table S1 Number and proportion of Clean Reads.
Table S2 Expression levels and basic information of all detected genes.
Table S3 DEGs identified in the comparison of SAM vs. ES, SAM vs. FS and SAM vs. MS.
Table S4 DEGs identified in the comparison of FS vs. MS, ES vs. MS.
Table S5 DEGs identified in the comparison of MS vs. SAM, MS vs. ES and MS vs. FS.
Table S6 Amplification primers of representative genes for qRT-PCR validation.
